# Effect of Milling Time on the Microstructure, Physical and Mechanical Properties of Al-Al_2_O_3_ Nanocomposite Synthesized by Ball Milling and Powder Metallurgy

**DOI:** 10.3390/ma10111232

**Published:** 2017-10-26

**Authors:** Meysam Toozandehjani, Khamirul Amin Matori, Farhad Ostovan, Sidek Abdul Aziz, Md Shuhazlly Mamat

**Affiliations:** 1 Materials Science and Characterization Laboratory, Institute of Advanced Technology, Universiti Putra Malaysia, 43400 Serdang, Selangor, Malaysia; Toozandejani.meysam@yahoo.com; 2 Department of Physics, Faculty of Science, Universiti Putra Malaysia, 43400 Serdang, Selangor, Malaysia; Sidek@upm.edu.my (S.A.A.); Shuhazlly@upm.edu.my (M.S.M.); 3 Department of Material Science and Engineering, Bandarabbas Branch, Islamic Azad University, Bandarabbas 7915893144, Iran; F.ostovan@gmail.com

**Keywords:** Al-Al_2_O_3_ nanocomposites, ball milling, microstructure, physical properties, mechanical properties

## Abstract

The effect of milling time on the morphology, microstructure, physical and mechanical properties of pure Al-5 wt % Al_2_O_3_ (Al-5Al_2_O_3_) has been investigated. Al-5Al_2_O_3_ nanocomposites were fabricated using ball milling in a powder metallurgy route. The increase in the milling time resulted in the homogenous dispersion of 5 wt % Al_2_O_3_ nanoparticles, the reduction of particle clustering, and the reduction of distances between the composite particles. The significant grain refining during milling was revealed which showed as a reduction of particle size resulting from longer milling time. X-Ray diffraction (XRD) analysis of the nanocomposite powders also showed that designated ball milling contributes to the crystalline refining and accumulation of internal stress due to induced severe plastic deformation of the particles. It can be argued that these morphological and microstructural variations of nanocomposite powders induced by designated ball milling time was found to contribute to an improvement in the density, densification, micro-hardness (*HV*), nano-hardness (*HN*), and Young’s modulus (*E*) of Al-5Al_2_O_3_ nanocomposites. *HV*, *HN*, and *E* values of nanocomposites were increased by ~48%, 46%, and 40%, after 12 h of milling, respectively.

## 1. Introduction

Application of Al-Al_2_O_3_ composites has been extended into the aerospace and automotive industries because of improved mechanical, tribological, and high-temperature properties [1,2,3,4,5]. Alumina (Al_2_O_3_) has been most widely used as ceramic reinforcement particulates in aluminum matrix composites (AMCs) due to the availability of different types, shapes, and sizes, its low cost and overall good properties [4,5,6,7,8,9].

During synthesizing Al-Al_2_O_3_ nanocomposites, like other metal matrix composites (MMCs), achieving a uniform dispersion of Al_2_O_3_ reinforcement particles and avoiding clustering of the Al_2_O_3_ reinforcement particles, particularly those at the nano-scale, would be a significant constraint. This is due to the fact that the enhancement of physical and mechanical properties is potentially decided by a uniform dispersion of Al_2_O_3_ reinforcement particles within the matrix. Then, reinforcing the aluminum matrix, particularly with Al_2_O_3_ nanoparticles, necessitates a proper technique to obtain a uniform dispersion of Al_2_O_3_ nanoparticles. Mechanical milling through ball milling enables uniform dispersion of reinforcements into the aluminum matrix, which avoids the agglomeration and clustering of the reinforcement particles [1,10,11].

According to the literature, Al-Al_2_O_3_ composites are successfully synthesized using the ball milling and powder metallurgy route. The homogenous dispersion of Al_2_O_3_ nanoparticles and improved mechanical behaviour of Al-Al_2_O_3_ nanocomposites is reported [1,2,4,7,9]. However, Al-Al_2_O_3_ nanocomposites are synthesized by the powder metallurgy route, but the powder metallurgy parameters, like densification, porosity, and physical dimensional change (PDC%) of bulk specimens, are not fully investigated. As a well-known fact, porosity is an inevitable part of the powder metallurgy (PM) product which, in turn, affects the final properties of composites.

In our previous work [1], the effectiveness of the milling process in providing homogenous dispersion of 1 wt % Al_2_O_3_ nanoparticles is reported. Therefore, the first objective of the investigation was to ensure the efficiency of designated ball milling processes on the incorporation of 5 wt % Al_2_O_3_ nanoparticles within a pure aluminum matrix. Secondly, the effect of designated milling time on morphology, microstructure, densification behaviour and, finally, mechanical behaviour of Al-5Al_2_O_3_ has been investigated.

## 2. Results and Discussion

**Figure 1:**
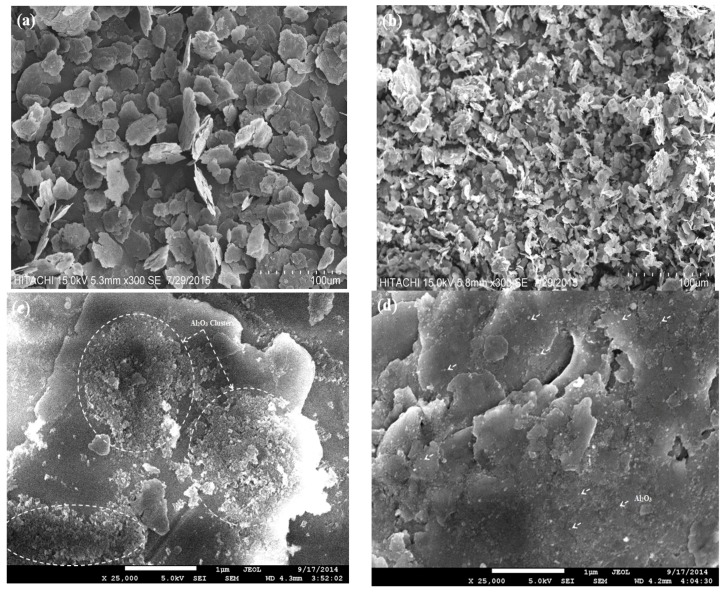
SEM micrographs of Al-5Al_2_O_3_ powder mixtures ball milled for (**a**) 2 h and (**b**) 8 h, showing the morphology of powder mixtures. FESEM micrographs of Al-5Al_2_O_3_ powder mixtures ball milled for (**c**) 0.5 h and (**d**) 8 h, showing the dispersion uniformity of Al_2_O_3_ powders.

It is well known that milling processes yield a significant change in the morphology of composite powders as a result of severe plastic deformation of the particles within the milling process [1,12]. Upon milling of Al-5Al_2_O_3_ nanocomposite powders, aluminum particles are welded and fractured until reaching a steady state where equiaxed particles are formed in the presence of Al_2_O_3_ nanoparticles. The morphology of Al-5Al_2_O_3_ nanocomposite powders at the beginning and the end of milling time is shown in Figure 1.

A short milling time of 0.5 h was found to have no significant effect on the morphology of powders except partial deformation of aluminum particles. At this stage, aluminum particles have kept their initial shape wherein the extreme clustering of Al_2_O_3_ nanoparticles could be observed. Figure 1c illustrates the presence of large amounts of Al_2_O_3_ clusters on the surface of aluminum particles as pointed out inside Figure 1c. As the milling time increased up to 2 h, cold welding of particles was activated and ductile aluminum particles started to flatten due to the high impact collision of the balls. The lamination of particles of soft aluminum was observed in some areas. Figure 1a shows that flake-shaped particles having large particle size are formed at this stage under deformation and cold welding of the particles. At the early stage of milling, up to 2, Al_2_O_3_ nanoparticles can rarely reside within the flattened aluminum particles and Al_2_O_3_ nanoparticle clusters are observed, but they are smaller and fewer in number than in powders milled for 0.5 h. Certainly, such microstructures have undesirable effects on mechanical properties. Flattening of the Al particles provides more sites for Al_2_O_3_ dispersion, which facilitates the uniform distribution of Al_2_O_3_ within the Al matrix.

The particles’ deformation was found to be more pronounced at longer milling times and a considerable change in morphology of nanocomposite powders was observable after 2 h of milling. Excessive work hardening and an increment of brittleness of soft aluminum powders activate the fracture mechanism; particles are fractured until reaching a steady state. Fracturing of work-hardened and brittle aluminum powders results in the formation of irregular-shaped powders and particles with smaller particle sizes until 8 h of milling. The progressive decrease in the particle size to Al_2_O_3_ particles encourage Al_2_O_3_ particles to be dispersed and embedded into the ductile aluminum particles more homogenously due to intense and repeated collision between the balls and the work-hardened powder. In fact, the finer matrix powder sizes, the more uniform the dispersion [13].

When the powder mixture reaches a steady state after 8 h, the particles’ morphology stabilizes and particles are in an equiaxed shape and certain size, which are randomly oriented as shown in Figure 1b. It is anticipated that in the steady state, the milling time is sufficient that no considerable change in the morphology of the powders is observable. At the steady state stage, Al_2_O_3_ nanoparticles are uniformly dispersed and the distance between Al_2_O_3_ nanoparticles is reduced; representing a tight bonding of the matrix and reinforcement at the steady state (Figure 1d). It can be inferred that the steady state can be achieved with the attainment of a full homogeneity of Al_2_O_3_ nanoparticles throughout the Al matrix [14]. A low amount of the Al_2_O_3_ nanoparticles can be observed because the remaining Al_2_O_3_ nanoparticles are embedded in the Al particles after reaching the steady state. At longer milling times, up to 12 h, the steady state is still predominated and milling has no considerable effect on the morphology of the powders, however, grain refinement is expected. The similar finding was found for Al-1 wt % Al_2_O_3_ in the authors’ previous study [1]. However, the deformation of the particles is more prominent in this study revealing the efficiency of the milling process in the presence of a larger content of Al_2_O_3_ nanoparticles. Alizadeh and Mirzaei Aliabadi [12] also reported a large Al_2_O_3_ content, and the contribution of the fracture mechanism increases and leads to a decrease in the particle size of the composite powder even with low milling time.

**Figure 2:**
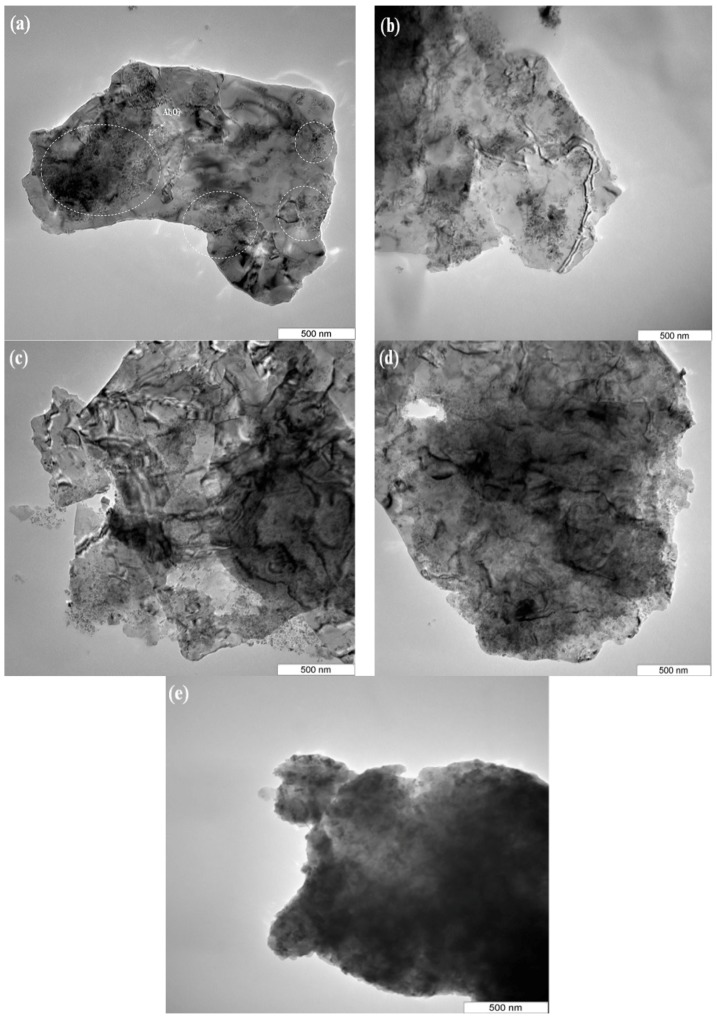
TEM micrograph of Al-5 wt % Al_2_O_3_ powder mixture milled for different times (**a**) 0.5 h; (**b**) 2 h; (**c**) 5 h; (**d**) 8 h; and (**e**) 12 h.

As can be observed in Figure 1c,d, Al_2_O_3_ nanoparticles which appear in large clusters (as highlighted in the Figure 1c) start to disperse within pure aluminum particles with a better homogeneity and smaller interparticle space by increasing the milling time. Figure 2 shows the dispersion uniformity of Al_2_O_3_ nanoparticles within the Al matrix particles more precisely at different milling times.

The dispersion of Al_2_O_3_ nanoparticles within the aluminum matrix increases by increasing the milling time until a well-homogenous dispersion is obtained at the steady state after 8 h of milling. A short milling time of 0.5 h has no noticeable effect on the dispersion of 5 wt % Al_2_O_3_ nanoparticles within the Al matrix. It can be seen that Al_2_O_3_ nanoparticles are heterogeneously dispersed on the surfaces of Al particles. Al_2_O_3_ nanoparticles appear in a clusters and most of the Al surface areas are not fully covered (Figure 2a). These independent clusters of Al_2_O_3_ nanoparticles, as shown by circles, are effectively broken up and start to disperse with better homogeneity throughout the Al matrix by increasing the milling time to 8 h. Although some clusters of Al_2_O_3_ particles can still be observed in some areas, they are smaller as compared with the short milling time. A homogeneous dispersion of Al_2_O_3_ nanoparticles throughout microstructure is observed after 8 h of milling (Figure 2d). It also seems that 8 h of milling is sufficient to disperse 5 wt % of Al_2_O_3_ nanoparticles within the aluminum matrix (Figure 2d). Milling times longer than 8 h results in no considerable changes in the dispersion of Al_2_O_3_ nanoparticles at the steady state. Figure 2e shows the adhesion of Al_2_O_3_ nanoparticles to the surface of aluminum particles after 12 h of milling, representing a good bonding between Al_2_O_3_ nanoparticles and aluminum particles. Ostovan et al. [8] also reported the homogenous dispersion of Al_2_O_3_ nanoparticles after 8 h of milling in the case of 1 wt % Al_2_O_3_. In fact, as milling proceeds, clusters of Al_2_O_3_ nanoparticle disappear, Al_2_O_3_ nanoparticles disappear throughout the matrix, and the distances between particles reduces [3,7,8,12,13,14].

**Figure 3:**
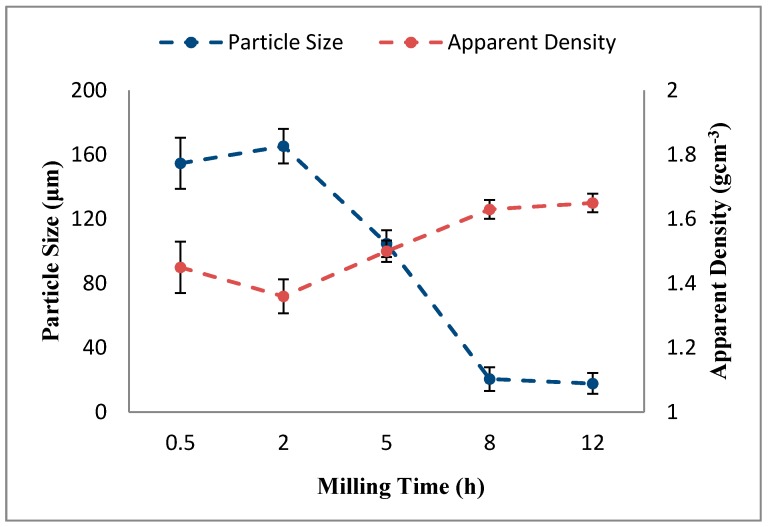
The variation of particle size and apparent density of Al-5Al_2_O_3_ nanocomposite powders as a function of the milling time.

The variation of particle size and apparent density of Al-5Al_2_O_3_ nanocomposite powders as a function of the milling time is shown in Figure 3. The average particle size Al-5Al_2_O_3_ nanocomposite powders increases in the first 2 h of milling and reaches a maximum of 165.3 (± 10.8) μm due to the predominance of cold welding. Cold welding, along with work-hardening of aluminum particles, leads to the formation of large particles with increased average particle size. This is similar to other composite systems as reported in the literature [8,13]. Razavi Tousi et al. [14] reported a continuous decrease in particle size of Al-Al_2_O_3_ composites in the presence of the high volume fraction of Al_2_O_3_ (20 vol %) where 20 vol % Al_2_O_3_ prevents cold welding from occurring. After 2 h of milling, the average particle size progressively decreases, representing the onset of the fracture mechanism, until 8 h of when steady state is reached. Fracturing of work-hardened and brittle aluminum powders results in the reduction of the average particle size of aluminum particles. At the steady state stage, a balance between cold welding and fracturing is achieved whereby the particle size is kept almost constant. Although the variation of average particle size in the steady state is not significant, it decreases from 20.58 (±7.34) μm to the lowest value of 17.82 (±6.45) μm at the end of ball milling. The results are in appropriate accordance with the morphology of the mixtures during milling where three different stages of the milling process could be identified.

Dissimilar to the variation of particle size, the apparent density of Al-5Al_2_O_3_ nanocomposite powders initially decreases at an early stage of milling, up to 2 h, then increases as the milling time increases, as shown in Figure 3. At early stages of milling, up to 2 h, the apparent density decreases due to the formation of large flake-shaped particles as a result of the cold welding of particles to a minimum of 1.36 (±0.05) g·cm^−3^. The apparent density of Al-5Al_2_O_3_ nanocomposite powders start to improve progressively after 2 h of milling, as ascribed by the fracturing of large particles and the formation of small, irregular shapes. The apparent density of Al-5Al_2_O_3_ nanocomposite powders vary in consistency with the variation in the morphology of Al particles, which varies from large flake-shaped particles at early stages of milling to more or less quasi-spherical particles having small particle sizes. Generally, flake-shaped particles indicate the worst packing properties of the powders, while spherical powders show better packing properties due to the relatively good mobility of particles and a lower tendency to form bridges [11,15,16]. Beyond 8 h of milling, in the steady state, formation of equiaxed particles improves the apparent density slightly to a maximum of 1.65 (±0.02) g·cm^−3^. The slight variation in apparent density in the steady state is attributed to the slight variation in the particle size of powders.

**Figure 4:**
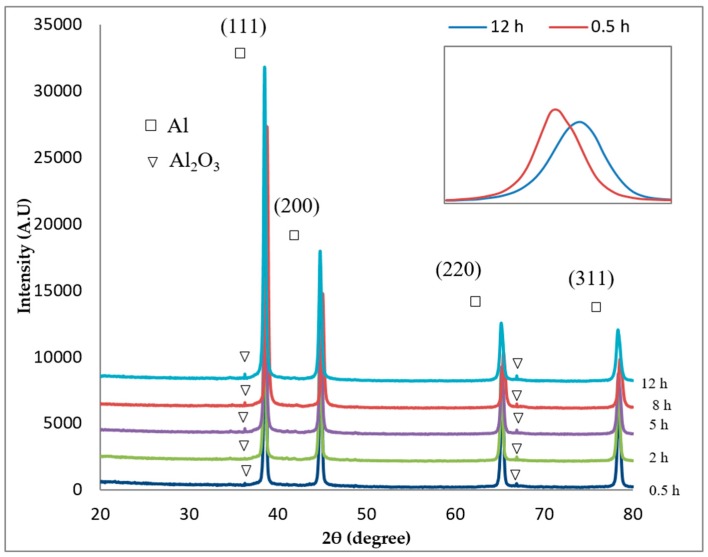
XRD diffraction patterns of Al-5Al_2_O_3_ nanocomposite powders milled for different times.

Figure 4 presents the X-ray diffraction patterns of Al-5Al_2_O_3_ nanocomposite powders ball milled for various times. There are four sharp detected diffraction peaks corresponding to the FCC structure of aluminum along with small Al_2_O_3_ peaks in XRD diffraction patterns of nanocomposite powders. The fewer Al_2_O_3_ peaks and their lower intensity in the diffraction pattern might be due to the low content of the Al_2_O_3_ phase and/or the fine size of the powder in the Al-5Al_2_O_3_ powder mixture [9,17]. Additionally, neither the presence of unwanted phases nor the impurity phases, such as Si, Fe, and Al_4_C_3_, was revealed in the XRD diffraction pattern. The results are inconsistent with previous findings by Zebarjad et al. [15] and Prabhu et al. [9]. The absence of impurities or unwanted phases indicates a clean interface between particles and the matrix. According to the literature, Al_2_O_3_ is so stable that no unwanted solid-state reaction occurs during the milling of aluminum and Al_2_O_3_ [18].

An increase in the milling time, up to 12 h, results in a definite shift in the position of the related peaks. An enlarged view of the shift position of the first peak corresponding to the crystalline plane of (111) of aluminum is shown in the right corner of Figure 4. The shift in the position of peaks may be due to the dissolution of minor alloying elements, impurities, and reinforcement particles in the lattice of the aluminum matrix during milling [16,18]. As is also shown in the right corner of Figure 4, the intensity of the first peak (111) in Al-5Al_2_O_3_ is decreased and its width broadened. By proceeding the milling time up to 12 h, peak intensities reduce and relative peaks become slightly broadened at the full-width at half-maximum (FWHM). In fact, the higher weight fraction of Al_2_O_3_ nanoparticles leads to higher peak intensities and the finer particle size results in the broadening of the peaks and lower peak intensities (smaller peak height) [9].

**Figure 5:**
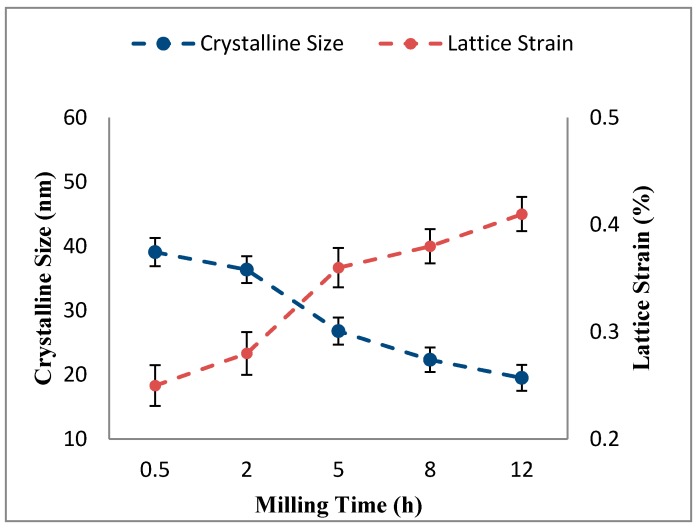
Variation of crystalline size and lattice strain of milled Al-5Al_2_O_3_ as a function of milling time.

Peak broadening of the ball-milled Al-5Al_2_O_3_ powders is corresponding to the crystalline size (or grain size) refinement and enhancement of lattice strain [2]. The Williamson-Hall method was used to analyse the relative peaks in order to relate the broadening in the XRD patterns to the crystalline size (*d*) and lattice strain (η) of Al-5Al_2_O_3_ nanocomposite powder mixtures. As shown in Figure 5, the crystalline size of aluminum particles generally decreases as the milling time increases, while lattice strain increases. The crystalline size of aluminum particles powders decreases from 39.10 (±2.2) nm at 0.5 h to 19.53 (±2) nm at 12 h of milling, while the lattice strain increases from 0.0026 (±0.019) to 0.0048 (±0.016) at the end of the milling process.

The significant variations in the crystalline size (or grain size) and in the lattice strain of aluminum particles is attributed to severe plastic deformation and grain size refinement occuring in the presence of Al_2_O_3_ nanoparticles within the milling process [19,20]. The main contribution to the decrement of crystalline size is the generation of dislocations as a result of local plastic deformation and accelerated work hardening of Al_2_O_3_ powders [14,21,22]. The interaction between hard and non-deformable Al_2_O_3_ nanoparticles and dislocations accelerates the grain-refining mechanism [16]. In the presence of Al_2_O_3_ nanoparticles, the Orowan bowing mechanism results in dislocation multiplication, as reported by Razavi Hesabi et al. [16]. By increasing the milling time, the dislocations are rearranged to a lower energy state and the low angle sub-boundaries are formed. The severe plastic deformation and generation of more dislocations at longer milling times leads to the increased misorientations between subgrains at their boundaries. Finally, low angle sub-boundaries turn into high angle boundaries and become grains with nano-scale sizes [17]. On the other hand, the interaction of Al_2_O_3_ nanoparticles with dislocations hinders the movement of dislocations resulting in an increase in dislocation density and an accumulation of stresses in the aluminum lattice. In addition, the distortion of the lattice structure and formation of other lattice defects, like vacancies and impurities within the milling process, results in the accumulation of internal stress in the aluminum lattice. Therefore, lattice strain gradually increases by increasing the milling time [14,23]. Ahmad et al. [18] have also reported the increase in the lattice strain as a result of less dissolution of alloying elements and reinforcement in the matrix at longer milling times.

**Figure 6:**
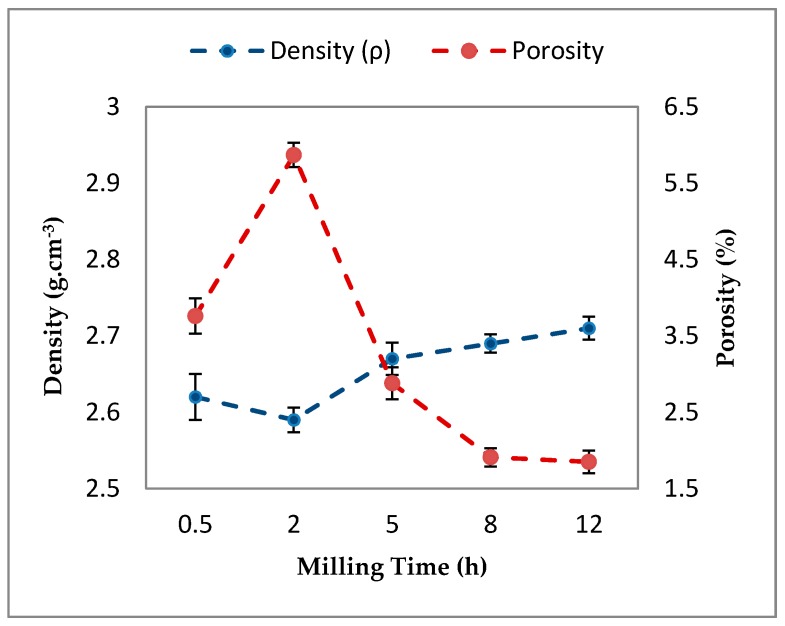
Variation of density and porosity of milled Al-5Al_2_O_3_ as a function of milling time.

Figure 6 presents experimental density and porosity of sintered Al-5Al_2_O_3_ nanocomposites with respect to milling time. The theoretical density of Al-5Al_2_O_3_ was also calculated according to the rule of mixtures and found to be 2.743 g·cm^−3^. The small increase of the theoretical density is due to the fact that the density of Al_2_O_3_ particles (3.95 g·cm^−3^) is slightly higher than that of aluminum (2.7 g·cm^−3^). It can be seen that the experimental density (*ρ*) increases from 2.62 (±0.03) to 2.71 (±0.015) g·cm^−3^ by increasing milling time from 0.5 h to 12 h. Although, there is a slight drop in the density values after 2 h of milling, as shown in Figure 6.

As a matter of fact, the sintered density is dependent on the morphology of the powders after milling, size, and distribution of the nano-reinforcement within the matrix, as well as compaction and sintering parameters [24]. Lower density at early stages of milling is due to the presence of aluminum agglomeration and Al_2_O_3_ clusters in the microstructure. Agglomeration and clustering of Al_2_O_3_ nanoparticles act as barriers that slow down the diffusion process which is needed for the sintering process. The increment of density is attributed to the better dispersion of Al_2_O_3_ nanoparticles within the matrix at longer milling times. At longer milling times, Al_2_O_3_ nanoparticles are dispersed homogeneously (Figure 2d,e), which accelerates the diffusion rate during sintering, as reported by [25]. These uniformly-dispersed Al_2_O_3_ particles are embedded in the grain boundaries and surfaces of aluminum particles and hinder the grain growth by a pinning mechanism, resulting in an increase in the density. On the other hand, more grain refining, which is showed as a reduction of the particle size resulting from longer milling time, further increases the diffusion rate.

**Figure 7:**
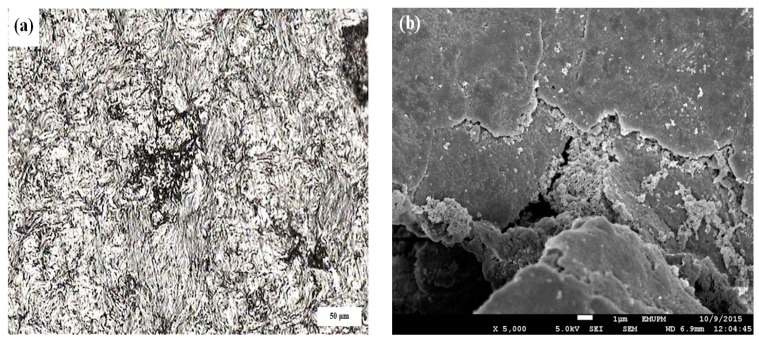
(**a**) Optical micrograph and (**b**) FESEM micrograph of the surface of Al-5Al_2_O_3_ nanocomposite milled for 2 h.

It can also be noticed that the values of experimental densities are lower than that of the theoretical densities indicating the presence of some amount of porosity in nanocomposite specimens, as shown in Figure 7. The amount of porosity in the sintered bulk Al-5Al_2_O_3_ nanocomposites is found to decrease significantly from 3.76% (±0.04) at 0.5 h to 1.85% (±0.03) at 12 h. As a well-known fact, porosity is an inevitable part of powder metallurgy (PM) product which is inversely proportional to density. At early stages of milling, nanocomposite specimens contain larger amounts of residual porosity as a result of clustered Al_2_O_3_ areas, which are the sites where cracks and porosities are formed, as reported by Zebarjad et al. [26]. Figure 7 shows the optical and FESEM micrographs of Al-5Al_2_O_3_ milled for 2 h, indicating the presence of porosities and voids formed on the polished surface of the composite. It can be seen that Al_2_O_3_ nanoparticles accumulate in the void formed on the surface of the nanocomposite (Figure 7b).

**Figure 8:**
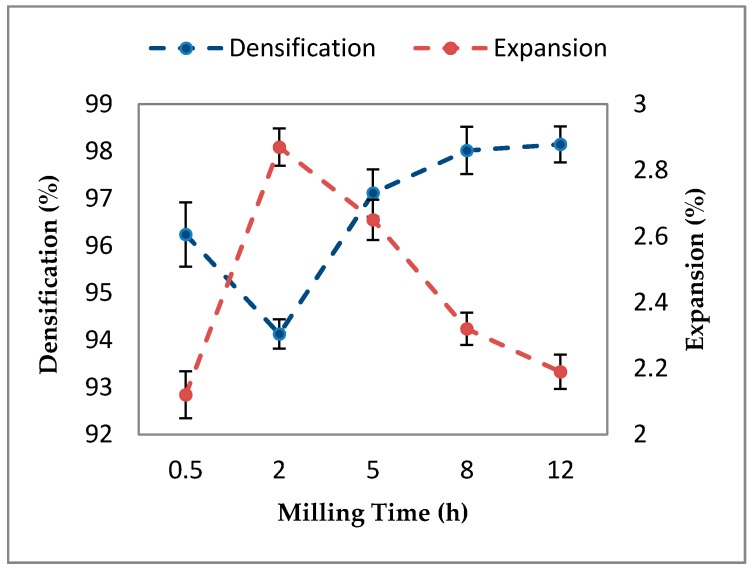
The variation of densification and expansion of Al-5Al_2_O_3_ composites as a function of milling time.

In addition, the densification mechanism shows a similar trend, as observed in the density. Densification decreases in the first 2 h of milling, reaching a minimum of 94.13%, and then increasing to 98.15% at 12 h of milling. The densification curve of the Al-5Al_2_O_3_ nanocomposite (Figure 8) is similar to the apparent density curve (Figure 3) which corresponds to the powder morphology and particle size variations.

As discussed earlier, at early stage of milling up 2 h, nanocomposite powders showing the worst packing properties due to formation of large flake-shape particles as a result of the cold welding of particles. Cold-welded and work-hardened powders decrease the pressing capacity of the specimens. The nanocomposites containing large particles cannot reach the full density. In contrast, particles with small sizes are easily densified rather than large particles under the same compaction conditions. Additionally, heterogeneous dispersion of Al_2_O_3_ nanoparticles and the formation of clustered regions at this stage lead to the lack of bonding between Al_2_O_3_ clusters and aluminum at the surface of the sintered composites. These microstructural features lead to higher plastic strain in clustered region and favors the formation of voids and cracks, and consequently-reduced mechanical properties. Uniform distribution of Al_2_O_3_ nanoparticles within the aluminum matrix at longer milling time and shorter distance between nanoparticles provide tighter bonding between Al_2_O_3_ nanoparticles and matrix, which results in the denser sintered composite. Conversely, as the bonding of particles is increased, the amount of porosities is decreased, so that a more dense part is obtained.

The variation of physical dimensional changes (or expansion) of Al-5Al_2_O_3_ nanocomposite specimens is shown in Figure 8. It can also be seen that dimensional expansion increases in the early stage of milling, up to 2 h. Nanocomposites milled for 2 h show the maximum expansion due to the cold welding of particles. At early stages of milling, the presence of cold-welded (work-hardened) particles and clustered Al_2_O_3_ areas hinder the diffusion of aluminum particles to form tighter bonds, resulting in greater expansion (grain growth) of specimens and a poor densification process [14]. As milling time proceeds, dimensional expansion decreases, indicating less grain growth during the sintering process as milling time increases (Figure 8). The dispersion homogeneity of Al_2_O_3_ nanoparticles increases by increasing the milling time and a better bonding of particles can be obtained resulting in less dimensional expansion of nanocomposite specimens during the sintering process. According to Mills [27], dimensional changes of composites are highly dependent to the compaction pressure, time and temperature of sintering, physical properties of the initial powder and the homogeneity of the reinforcement powder.

**Figure 9:**
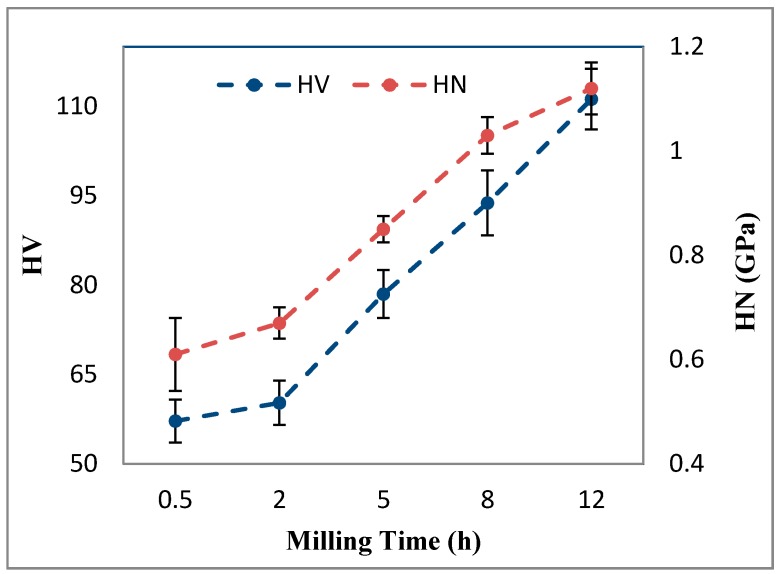
Variation of micro-hardness (*HV*) and nano-hardness (*HN*) of Al-5Al_2_O_3_ composites as a function of milling time.

The variation of micro-hardness (*HV*) and nano-hardness (*HN*) of Al-5Al_2_O_3_ nanocomposites as a function of milling time is presented in Figure 9. It is seen that as milling time proceeds, *HV* values of Al-5Al_2_O_3_ nanocomposite specimens increase. However, it can be noticed that the improvement of hardness at early stages of milling, up to 2 h, is not significant, whereas it is more profound at longer milling times. *HV* values were found to increase sharply, as it is the expected trend, from 60.25 (±3.6) *HV* after 2 h of milling to a maximum value of 111.20 (±5.1) *HV* at the end of milling, corresponding to an improvement of about 46%. Similarly, *HN* values of nanocomposite specimens increases by increasing the milling time. The enhancement of *HN* values in the milling time longer than 2 h is also noticeable. Al-5Al_2_O_3_ nanocomposites milled for 12 h showed *HN* of about twice (1.12 GPa) that of the *HN* of nanocomposites milled for 0.5 h (0.61 GPa), indicating the profound effect of the milling process in the improvement of the mechanical behaviour. The results are in agreement with previous studies where hardness of Al-5Al_2_O_3_ nanocomposite increases by increasing milling process [17,26,28,29].

In both micro- and nano-hardness measurements, the increment f *HV* and *HN* values of milled Al-5Al_2_O_3_ nanocomposites is attributed to the presence of Al_2_O_3_ nanoparticle reinforcements and microstructural variation within milling process of Al-5Al_2_O_3_ nanocomposite as also reported by Zebarjad et al. [26] and Mazilkin et al. [28]. Recall that, at early stages of milling, up to 2 h, cold welding of particles is predominated and hardening is expected because of work-hardening of particles while milling. Within the milling process of Al-5Al_2_O_3_ nanocomposites, severe local deformation of Al particles in the vicinity of Al_2_O_3_ nanoparticles result in the cold-working and grain-refining of Al particles which consequently increases the hardness of the powders. In addition, at this stage, Al_2_O_3_ nanoparticles are heterogeneously dispersed and mostly clustered, wherein the distance between composite particles is large (Figure 1a and Figure 2a), resulting in poor sintering. During sintering processes, Al_2_O_3_ nanoparticle clusters interrupt the diffusion bonding of particles, resulting in large amounts of residual porosity in bulk nanocomposites and, consequently, poor consolidation. Then, this microstructural state contributes to the lower hardness of nanocomposites due to poor sintering in the presence of Al_2_O_3_ clusters.

After 2 h of milling, both *HV* and *HN* values increase when dispersion of Al_2_O_3_ nanoparticles becomes more homogeneous and significant grain size refining occurs. Enhancement of the homogeneous dispersion of Al_2_O_3_ nanoparticles by increasing the milling time decreases the distance between nanocomposite particles so that further sintering processes will result in better bonding and, consequently, enhanced strength properties. Homogeneity of Al_2_O_3_ nanoparticles in the sintered composites has been directly related to the homogeneity of nanoparticles before sintering [1,26,29]. These small Al_2_O_3_ nanoparticlesm which have a short distance from each other, act like a barrier; thus, grain growth will be postponed in milled nanocomposite specimens [15]. By increasing the milling time, the average particle size of aluminum particles profoundly decreases, which results in a significant increase in the hardness of the matrix particles. The Al_2_O_3_ nanoparticles, by pinning the grain boundaries, can also hinder the grain growth mechanism and improve the strengthening mechanism. Moreover, the pure Al-5Al_2_O_3_ nanocomposites are comprehensively strengthened by the generation of high-density dislocations and the formation of fine sub-grains within the milling process, as mentioned earlier. At longer milling times, severe particle deformation, however, yields a homogeneous dispersion of Al_2_O_3_ nanoparticles within the Al matrix, but introduces strain hardening to the matrix particles through the generation of a large amount of high-density dislocations [28,29]. In fact, dispersed Al_2_O_3_ nanoparticles inhibit dislocation movement resulting in the generation of a large amount of high-density dislocations around the particles. Grain refinement is accelerated when a high dislocation density is present in the microstructure, which contributes to formation of extensive fine sub-grains in the matrix. Then, grain refinement through sub-grain formation strengthens the nanocomposites [29,30,31]. Different orientations of adjacent sub-grains and the high lattice disorder characteristic of the boundary regions hinder dislocation movement in a continuous slip plane and consequently harden the nanocomposites [32].

**Figure 10:**
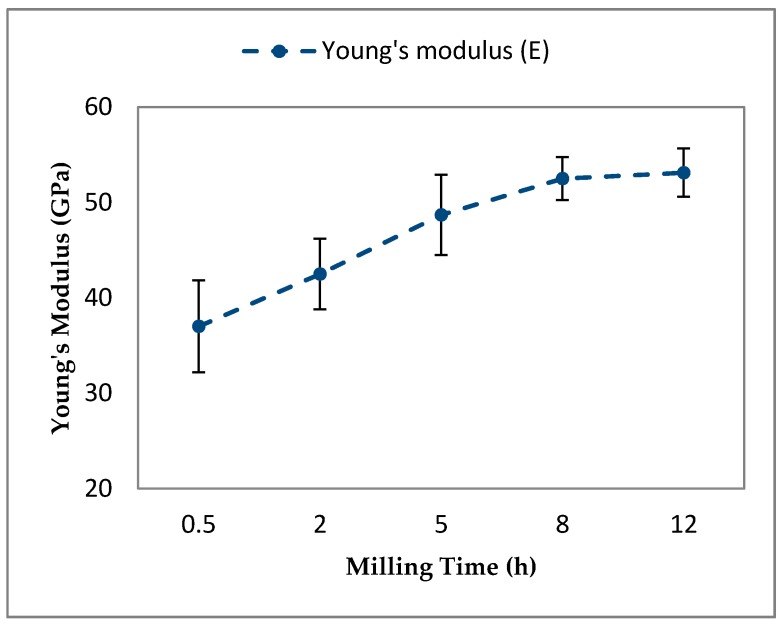
The variation of E values of Al-5Al_2_O_3_ nanocomposites as a function of milling time.

An upward trend in the variation of *E* values of Al-5Al_2_O_3_ nanocomposites was also obtained by increasing the milling time up to 12 h (Figure 10). Nanocomposites milled for a short milling time of 0.5 h have the lowest E values of 37.1 (±4.81) GPa. The *E* values of nanocomposites increase linearly to 52.50 (±2.24) GPa after 8 h of milling at a steady state. The upward increasing trend of the Young’s modulus extends to the longer milling time of 12 h in the steady state stage where the maximum value of the Young’s modulus value of 53.12 (±2.53) GPa, representing an increase in *E* values of about 28%.

**Figure 11:**
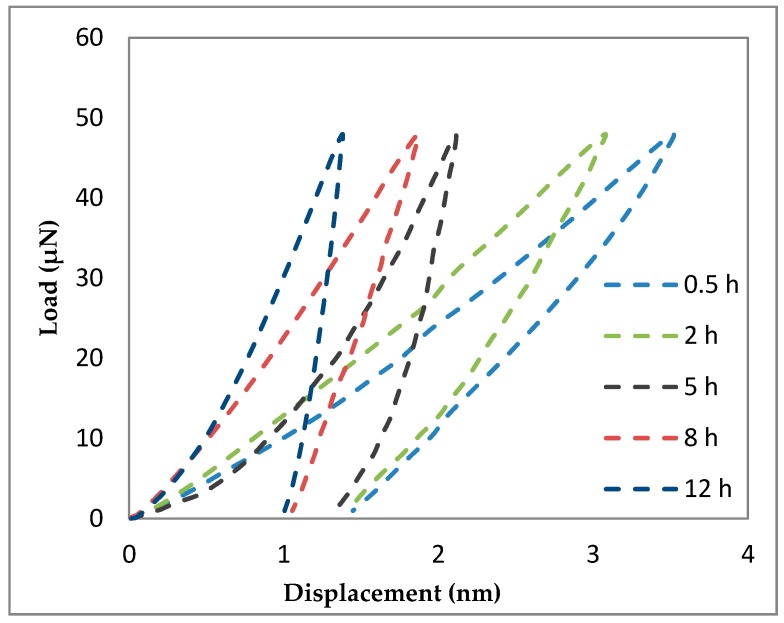
Load–displacement curves of Al-5Al_2_O_3_ nanocomposite at different milling time.

A representative load-displacement curve of Al-5Al_2_O_3_ with respect to milling time at a peak load of 100 μN is shown in Figure 11. In nanoindentation measurement, elastic modulus is a function of the slope of the unloading curve. It can be seen that nanocomposites milled for the shortest time of 0.5 h shows the highest displacement, attributed to the lowest Young’s modulus. Conversely, nanocomposites milled for the longest milling time of 12 h shows the lowest displacement, indicating the maximum Young’s modulus under the same indentation conditions. The lower displacement or lower indention depth in nanoindentation measurements represents the strengthening of the matrix in the presence of Al_2_O_3_ nanoparticles [33]. Then, the load-displacement curve of Al-5Al_2_O_3_ shifts toward the left and lower displacements by increasing the milling time due to strengthening of the matrix.

Similar to *HV* and *HN*, the variation of the Young’s modulus is attributed to the state of dispersion of Al_2_O_3_ nanoparticles and the grain refinement of particles within the milling process. At shorter milling times, heterogeneous dispersion and clustering of Al_2_O_3_ nanoparticles and larger particle sizes of particles contributes to the lower Young’s modulus of Al-5Al_2_O_3_ nanocomposites. By increasing the milling time, the Young’s modulus of Al-5Al_2_O_3_ nanocomposites increases, owing to a more homogenous dispersion of Al_2_O_3_ nanoparticles and along with the grain refining of aluminum particles.

As a matter of fact, the Young’s modulus, or resistivity of material to being elastically deformed, of powder metallurgy is significantly affected by the amount of porosity and cracks present in the sintered compacts. The presence of porosities and cracks in the sintered compacts noticeably lowers the Young’s modulus of MMCs [9,34]. Heterogeneous dispersion and clustering of Al_2_O_3_ nanoparticles result in the formation of more porosity and cracks after the sintering process. Resultant residual porosities cause stress concentrations and unexpected fractures, thus, lowering the strength. In contrast, homogeneous dispersion Al_2_O_3_ nanoparticles provide a better bonding of particles during the sintering process. The homogeneously-dispersed Al_2_O_3_ nanoparticles and very short distances between Al_2_O_3_ nanoparticles prevent crack extension and their interconnection and reduce stress concentration, therefore, enhancing the strength properties of the nanocomposites.

Finally, it should be mentioned that there are some mechanisms which may simultaneously be activated that enhance the strength properties of the Al-5Al_2_O_3_ nanocomposites like other MMCs. These mechanisms are include Orowon strengthening as a result of the uniform dispersion of Al_2_O_3_ particles, solid solution hardening, work hardening as a result of the strain misfit between the reinforcement and matrix particles, grain and substructure strengthening following the classical Hall-Petch relationship, quench hardening due to the dislocations generated to accommodate the differential thermal contraction between reinforcement and matrix particles, and thermal mismatch.

## 3. Materials and Methods

**Figure 12:**
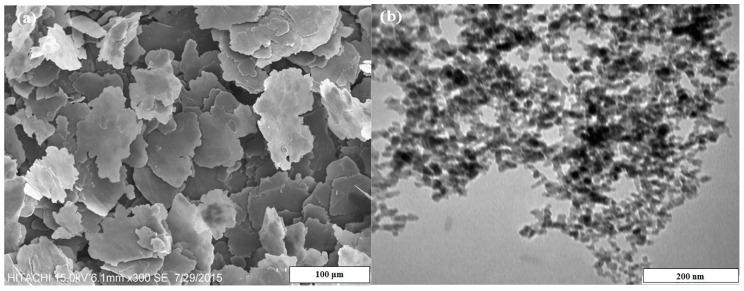
Surface morphology of the as-received (**a**) pure Al and (**b**) Al_2_O_3_ powders.

Flake shape of fine aluminum (Al) powder supplied by Merck (Merck KGaA, Darmstadt, Germany, 99% purity, 200 μm) and spherical nano-alumina (Al_2_O_3_, Sigma Aldrich, St. Louis, MO, USA, 200 nm) were used as a raw material to fabricate Al-5 wt % Al_2_O_3_ nanocomposites. Figure 12 shows the surface morphology of the as-received powders. The as-received atomized aluminum powders are predominantly equiaxed and irregular in shape.

Nanocomposite powder mixtures containing 5 wt % Al_2_O_3_ nanoparticles were prepared along with 2 wt % stearic acid as a process control agent (PCA) to prevent excessive cold welding of the particles within milling. However, stearic acid stearic acid will then be vaporized during sintering before the temperature of 361 °C [21]. The nanocomposite powder mixtures were placed in a tungsten carbide jar with the ball-to-powder weight ratio (BPR) of 8:1. Argon (Ar) was purged into the confined and sealed jar before milling to carry out the ball milling under an Ar atmosphere. A planetary ball milling machine (PM 100, Retsch, Haan, Germany) was used to ball mill Al-5 wt % Al_2_O_3_ nanocomposites powder. The powder mixtures were ball milled for various milling time from 0.5 to 12 h. The rotational speed of the ball milling machine was maintained constant at 300 rpm. Afterwards, nanocomposite powders were compacted using a uniaxial cold press at a pressure of 150 MPa. Sampling was accurately done in the glove box under argon atmosphere to avoid oxidation and obtain sound bulk nanocomposite specimens with free, or the least amount, of pores. Nanocomposite compacts were finally sintered at 580 °C for 45 min under an Ar atmosphere.

The morphology and dispersion uniformity observation of ball-milled powders was initially performed using scanning electron microscopy (SEM Hitachi S-3400, Tokyo, Japan), and transmission electron microscopy (TEM, LEO 912AB, Zeiss, Germany). The surface of the bulk nanocomposites were prepared by following standard metallographic techniques. Field emission scanning electron microscopy (FESEM, 7600F, JEOL, Tokyo, Japan) and a Leica light microscope (Leica, Wetzlar, Germany) equipped with a Q_win_ image analyser was used to observe the carefully-polished surface of specimens in the case of porosities.

The particle size of composite powders was measured in each step of milling with MasterSizer analyser2000 (Malvern Instruments Ltd, Worcestershire, UK), using laser light diffraction. The mean size of the volume-weighted particles was reported. Phase analysis of nanocomposite powders within the milling process was performed by a Shimadzu XRD-6000 (Shimadzu Corporation, Osaka, Japan) using 0.1542 nm CuK_α_ 181 radiation at 4.8 kW with the voltage of 40 kV and a current of 120 mA. Individual phases were identified by matching the characteristic XRD peaks against ICCD data. Present peaks in the XRD patterns of nanocomposite powders were indexed using Xpert Highscore plus software (PANalytical, Almelo, The Netherlands). Crystalline size (d) and lattice strain (η) of aluminum particles were determined using the Williamson and Hall method and based on the following equation:

(1) $$b\;\cos\theta =\frac{0.9\;\lambda }{d}+2\eta \;\sin\;\theta$$

where *b*, θ, λ, *d*, and η are the full-width of the peak at half maximum intensity (FWHM), the Bragg angle, X-ray wavelength used, crystalline size, and lattice strain of the nanocomposite powders, respectively [35].

The experimental density of the nanocomposites was measured was measured according to the Archimedes principle at room temperature:

(2) $$\rho =\frac{W_{a}}{W_{a}-W_{b}}\times \rho_{b}$$

where *W_a_* is the weight of specimens in air, *W_b_* the weight of specimens in buoyant and ρ*_b_* is the density of the buoyant, respectively. The average of three density measurement was reported as the density value. The theoretical density of the nanocomposites was obtained according to using the rule of mixture. According to the rule of mixture if reinforcement particle of volume fraction ν*_r_* is now added to aluminum matrix, the effective density of the composite, ρ*_c_*, is given by:

(3) $$\rho_{c}=\rho_{r}\nu_{r}+\rho_{std}\left(1-\nu_{p}\right)\left(1-\nu_{r}\right)$$

where $\rho_{r}$ and $\rho_{std}$ are the density of the reinforcement particles and standard pore free density of the matrix material, respectively [36]. The amount of porosities of the nanocomposites was measured from the difference between the expected and the observed density of each sample.

The physical dimensional changes (PDC) of the nanocomposite specimens were determined according to ASTM B610 standard using the following equation:

(4) $$\%\;PDC=\frac{D_{s}-D_{g}}{D_{g}}\times 100$$

where Dg and Ds are the dimension of the samples before and after sintering, respectively. A positive % PDC value indicates the growth of the samples, and a negative % PDC values reflects shrinkage of the specimens [37].

The microindentation hardness was determined using a Mitutoya HV-112 Vickers hardness measurement machine (Mitutoyo Corporation, Kanagawa, Japan). Ten Vickers indents at a load of 100 gf and a dwell time of 10 s was applied on the polished cross-section of bulk nanocomposite specimens and the average reported for *HV* values. Nanoindentation was performed by a Micro Materials Nanotest™ indenter (Micro Materials, Wrexham, UK) equipped with a standard Berkovich geometry tip indenter, at a maximum load of 50 μN and dwell time of 5 s applied to 20 different locations of the polished surface of the nanocomposites. The indentations were made at constant loading rates of 0.5 μN/s until reaching the maximum load of 50 μN and before being unloaded gradually to zero. The *HN* and *E* of values of the nanocomposites were calculated according to the Oliver–Pharr method [38]. The average values of *HN* and *E* values of a total of 20 indents made at different locations and peak load were reported.

## 4. Conclusions

This study was conducted to investigate the effect of milling time on morphology, microstructure, and consequent physical and mechanical behaviour of pure Al-5Al_2_O_3_. It was found that increasing the milling time contributes to the homogenous dispersion of 5 wt % Al_2_O_3_ nanoparticles, the reduction of particle clustering, and the reduction of distances between the composite particles. The increase of the milling time also leads to grain refining due to induced severe plastic deformation of the particles within milling, as shown by particle size reduction. These morphological and microstructural variations at longer milling time enhance the *HV*, *HN*, and *E* values of Al-5Al_2_O_3_ nanocomposite by ~48%, 46%, and 40%, after 12 h of milling, respectively. Experimental density, densification, and physical dimensional changes of nanocomposites were also found to increase with an increase in the milling time. The enhanced physical and mechanical behaviour of Al-5Al_2_O_3_ nanocomposites is attributed to the increase in the milling time where a homogenous dispersion of Al_2_O_3_ nanoparticles was obtained along with grain refining, whereas agglomeration of Al particles and clustering of Al_2_O_3_ at shorter milling times contribute to the formation of porosities and cracks which weaken the sintering process and, thus, finally, the physical and mechanical behaviour of Al-5Al_2_O_3_ nanocomposites.
